# Comparative Evaluation of Visual Outcomes and Patient Satisfaction After Eyecryl SERT Versus TECNIS Eyhance Intraocular Lens Implantation in Cataract Patients

**DOI:** 10.7759/cureus.102369

**Published:** 2026-01-27

**Authors:** Sri Ganesh, Malvika Krishnan, R. Prakhya, Bhargav Joshi

**Affiliations:** 1 Department of Cataract and Refractive Surgery, Nethradhama Super Speciality Eye Hospital, Bengaluru, IND; 2 Department of Clinical Affairs, Biotech Vision Care Pvt. Ltd., Ahmedabad, IND

**Keywords:** cataract extraction, extended depth of focus, intra-ocular lenses, presbyopia correction, senile cataract

## Abstract

Background

Intraocular lenses (IOLs) that extend a single focal point are designed to improve intermediate vision while maintaining the optical clarity of traditional monofocals. Unlike multifocal designs, they aim to reduce visual disturbances such as glare and halos, offering a more comfortable visual experience. These lenses are particularly beneficial for modern tasks like computer use, cooking, or dashboard viewing, where intermediate vision plays a critical role. This study is part of a larger prospective investigation comparing visual outcomes, patient satisfaction, and safety of the Eyecryl SERT and TECNIS Eyhance IOLs after cataract surgery.

Methods

This prospective, comparative clinical study included patients undergoing cataract surgery who were implanted with either Eyecryl SERT or TECNIS Eyhance IOLs. After obtaining informed consent, preoperative screening and clinical assessments were performed. Postoperative follow-ups were conducted on Day 1, Week 2, and Month 6. Outcome measures included binocular and monocular uncorrected and distance-corrected intermediate visual acuity (DCIVA) at 66 cm under photopic conditions, contrast sensitivity, defocus curve, reading speed, patient-reported satisfaction, posterior capsule opacification (PCO), and adverse event rates.

Results

The study included 120 eyes from 60 participants, with 30 subjects in each IOL group, all of whom completed follow-up visits. At six months, Eyecryl SERT demonstrated superior intermediate visual outcomes compared to TECNIS Eyhance, with significant improvements in binocular DCIVA at 66 cm (0.02 ± 0.06 vs. 0.08 ± 0.07 logMAR; p < 0.0001) and monocular DCIVA at 66 cm (0.05 ± 0.09 vs. 0.13 ± 0.08 logMAR; p < 0.0001). Monocular UCIVA also improved in the Eyecryl SERT group compared to TECNIS Eyhance (0.08 ± 0.10 vs. 0.18 ± 0.07 logMAR; p < 0.0001). Eyecryl SERT demonstrated better contrast sensitivity at all spatial frequencies (p < 0.0001) and higher reading speeds (near: 184.4 ± 23.12 vs. 174.8 ± 17.53 words per minute; p = 0.0074). Defocus curve performance was superior for Eyecryl SERT at -1.0 D and -1.5 D (p = 0.05 and p = 0.009). No PCO or adverse events were reported.

Conclusions

The Eyecryl SERT IOL demonstrated superior intermediate visual acuity, contrast sensitivity, and reading performance compared with the TECNIS Eyhance over a six-month follow-up period, without any associated safety concerns. These results suggest that Eyecryl SERT may provide a functional advantage for tasks requiring intermediate vision, potentially enhancing patient satisfaction in daily activities. However, further long-term studies with larger and more diverse populations are needed to validate these findings and assess their broader clinical applicability.

## Introduction

Cataract surgery has evolved significantly over the past decades, transitioning from a purely vision-restorative procedure using monofocal intraocular lenses (IOLs) to a form of refractive surgery aimed at achieving spectacle independence. Technological advances have enabled the development of premium IOLs, including toric and presbyopia-correcting designs such as multifocal and accommodating IOLs, which address patients’ increasing demand for functional vision across multiple distances [1].

Multifocal IOLs, through diffractive or refractive optics, are designed to provide additional focal points for near and intermediate vision. While they improve spectacle independence, these lenses are frequently associated with visual disturbances such as halos, glare, and starbursts, particularly under low-light conditions [2,3]. Furthermore, they often result in reduced contrast sensitivity in mesopic and scotopic environments, limiting their suitability for patients requiring optimal night vision [4].

Accommodative IOLs, which aim to simulate physiological accommodation by shifting optical power via anterior-posterior lens movement or changes in lens shape, have demonstrated inconsistent long-term results [5]. Their performance is often compromised by posterior capsule opacification (PCO), capsular contraction, and lens tilt, leading to asymmetric vaulting and unstable refractive outcomes. Consequently, accommodative IOLs have largely fallen out of favor in routine clinical practice due to these limitations in both efficacy and predictability [5,6].

In contrast, extended depth-of-focus (EDOF) IOLs and advanced monofocals have emerged as compelling alternatives, offering several advantages over both multifocal and accommodative lenses. Both aim to provide a broader range of vision while minimizing the visual disturbances often associated with multifocal optics, such as glare, halos, and reduced contrast sensitivity. EDOF IOLs are designed to achieve a clinically meaningful extension of vision, defined by an increase of at least 0.5 D in depth of focus at a visual acuity level of 0.2 logMAR (20/32) compared with standard monofocal lenses, as outlined in American National Standards Institute (ANSI) Z80.35 criteria. Enhanced monofocal IOLs, while not fully meeting ANSI Z80.35 criteria, still provide a measurable improvement in intermediate vision over conventional monofocals while preserving excellent distance vision and optical quality [7,8].

The Eyecryl SERT IOL (Biotech Vision Care Pvt. Ltd., Ahmedabad, India) is a hydrophobic, aspheric EDOF IOL with an aspheric surface leveraging proprietary Monomore^®^ technology. This design features optimized optical zones for extended vision and asymmetric power distribution to reduce pupil dependency while maintaining low levels of photic phenomena, similar to standard monofocal IOLs [9]. The TECNIS SIMPLICITY™ Delivery System Model DIB00 (Johnson & Johnson Vision, Irvine, CA, USA) contains the TECNIS Eyhance™ IOL, a one-piece, foldable, posterior chamber lens whose optical design provides a modest increase in depth of focus. The TECNIS Eyhance™ IOL is intended to slightly extend depth of focus compared to its monofocal analogue, the TECNIS™ 1-Piece IOL, Model ZCB00 [10].

The current study is a prospective, comparative clinical investigation evaluating the efficacy, safety, and patient satisfaction associated with Eyecryl SERT and the established TECNIS Eyhance IOL following cataract surgery.

## Materials and methods

Study design and patients

This prospective, comparative clinical study was conducted between August 2022 and May 2024 with the approval of the Institutional Ethical Committee of Nethradhama Super Speciality Eye Hospital, Bengaluru, India, and in accordance with the tenets of the Declaration of Helsinki. After receiving a complete explanation of the study, all patients provided written informed consent. This trial was registered with the Clinical Trials Registry - India (CTRI) under the identification number CTRI/2022/05/042838. This study forms part of a broader prospective evaluation of two IOLs and focuses specifically on intermediate visual performance and associated functional outcomes.

Patients were enrolled consecutively, and lens selection reflected routine clinical practice and availability during the study period. Inclusion criteria were cataract patients aged 22 years or older with best-corrected visual acuity (BCVA) of 20/40 or worse, anticipated postoperative BCVA better than 20/30, and preoperative keratometric astigmatism ≤ 0.75 D. Exclusion criteria included systemic diseases or medications that could increase operative risk or confound outcomes (e.g., tamsulosin-related floppy iris syndrome), ocular conditions predisposing to complications, previous intraocular or corneal surgery, degenerative visual disorders (e.g., macular degeneration), or factors affecting lens centration. Pregnant or lactating individuals and those with hormonal fluctuations affecting refraction were also ineligible. Additionally, subjects requiring retinal laser treatment were excluded if preclinical testing suggested an increased risk of light scatter.

Preoperative examination

All patients underwent a comprehensive preoperative ophthalmologic evaluation, including measurement of uncorrected and corrected visual acuity at distance and intermediate using the ETDRS chart (Precision Vision, La Salle, IL, USA). A detailed slit-lamp biomicroscopy and dilated fundus examination were performed to assess ocular health. Intraocular pressure was measured using Goldmann applanation tonometry. Keratometry, axial length, and anterior chamber depth were measured using the Pentacam. IOL power calculation was performed with IOL Master 700 optical biometry using the Barrett Universal II formula.

Study IOLs

The Eyecryl SERT IOL (preloaded, hydrophobic acrylic foldable, single-piece posterior chamber IOL) is designed for surgical implantation as a replacement for the natural crystalline lens. The lens is made from medical-grade hydrophobic material with less than 5% water content. The TECNIS Eyhance™ IOL, delivered using the TECNIS Simplicity™ Delivery System (Model DIB00), is an ultraviolet-light-absorbing posterior chamber IOL intended to replace the optical function of the natural crystalline lens within the capsular bag (Table 1).

**Table 1:** Description of IOL specifications IOL, intraocular lens

Parameter	Eyecryl SERT IOL	TECNIS Eyhance™ IOL
Material	Hydrophobic acrylic containing a natural chromophore	Soft, foldable hydrophobic acrylic with a covalently bound UV absorber
Optic type	Single-piece, 360° square edge with refractive aspheric optic	1-piece IOL, ProTEC frosted, continuous 360° posterior square edge
Optic diameter	6.00 mm	6.00 mm
Overall diameter	13.00 mm	13.00 mm
Refractive index	1.524	1.47 at 35 °C
Diopter range	+7.0 D to +30.0 D (0.5 D steps)	+5.0 D to +34.0 D (0.5 D increments)
Delivery system	Preloaded delivery system	TECNIS Simplicity system

Surgical procedure

All surgeries were performed by a single experienced surgeon (SG) using a standardized technique. Topical anesthesia with 0.5% proparacaine eye drops and intracameral 2% lidocaine was administered to ensure patient comfort. A 2.8 mm temporal biplanar micro-incision was created, followed by phacoemulsification using the Centurion^®^ Vision System (Alcon Laboratories, Inc., Fort Worth, TX, USA). IOL implantation was performed according to standard clinical protocols.

Continuous curvilinear capsulorhexis was created either manually using a 26G needle or with femtosecond laser assistance. The proportion of femtosecond-assisted cases was comparable between both IOL groups, targeting a capsular opening of 5.0-5.5 mm. Preoperatively, patients received mydriatic and anti-inflammatory eye drops, including 0.5% tropicamide with phenylephrine hydrochloride and 0.1% flurbiprofen. During surgery, Aurovisc (Aurolab, Madurai, India) ophthalmic viscoelastic devices were used to maintain anterior chamber stability and facilitate IOL implantation.

Outcome and assessment

Postoperative assessments were conducted at Day 1, Week 2, and Month 6. Intermediate visual acuity was measured at 66 cm, both monocularly and binocularly, during the two-week and six-month visits. The primary endpoint was binocular distance-corrected intermediate visual acuity (DCIVA) at 66 cm. Secondary endpoints included uncorrected intermediate visual acuity (UCIVA), nondirected optical/visual symptoms assessed at Day 1 and Month 6, and other six-month evaluations, which included PCO assessment, a subjective vision-related quality-of-life questionnaire, contrast sensitivity measured using the CSV-1000, reading performance using the Radner Chart, and defocus curve testing.

Statistical analysis

The sample size was determined based on the primary efficacy endpoint: binocular DCIVA at 66 cm under photopic conditions. Assuming a mean difference of 1.0 line between groups, an SD of 1.2 lines, 85% power, 5% alpha, and a 1:1 allocation ratio, a total of 60 subjects (30 per group) were planned for enrollment, accounting for a 10% dropout rate, to ensure 52 evaluable subjects (26 per group).

Continuous data were summarized using descriptive statistics, including number of eyes (n), mean, SD, median, minimum, and maximum. Categorical data were summarized using frequency counts (n) and percentages (%). Depending on the data distribution, parametric tests (two-sample or paired t-test) or nonparametric tests (Mann-Whitney U or Wilcoxon signed-rank test) were applied for inter- and intra-group comparisons. All statistical tests were conducted at the 95% CI, and a p-value ≤ 0.05 was considered statistically significant. Statistical analysis was performed using SAS software version 9.4 (SAS Institute Inc., Cary, NC, USA).

## Results

The study included a total of 120 eyes from 60 participants, with 30 subjects in each IOL group. All participants completed the follow-up visits. The demographic details are summarized in Table 2.

**Table 2:** Demographic and baseline characteristics of the study population IOL, intraocular lens

Characteristic	Eyecryl SERT IOL (n = 30)	TECNIS Eyhance™ IOL (n = 30)	p-Value
Age (years), mean ± SD; range	67.3 ± 8.0; 48-89	65.9 ± 7.4; 45-81	0.484
Gender, n (%)			
Male	15 (50.0)	18 (60.0)	0.603
Female	15 (50.0)	12 (40.0)	0.603
Subjective questionnaire, mean ± SD	74.45 ± 3.28	73.82 ± 5.12	0.625

Visual acuity

DCIVA at 66 cm

At 66 cm, binocular DCIVA showed significant improvement in the Eyecryl SERT group, increasing from 0.03 ± 0.06 logMAR at two weeks to 0.02 ± 0.06 logMAR at six months (mean change: -0.01 ± 0.03 logMAR; p = 0.031). In contrast, the TECNIS Eyhance group remained stable at 0.08 ± 0.07 logMAR at both time points (mean change: 0.00 ± 0.00 logMAR; p = 0.000). Inter-group comparison at six months revealed a statistically significant advantage for Eyecryl SERT (p < 0.0001).

**Figure 1:**
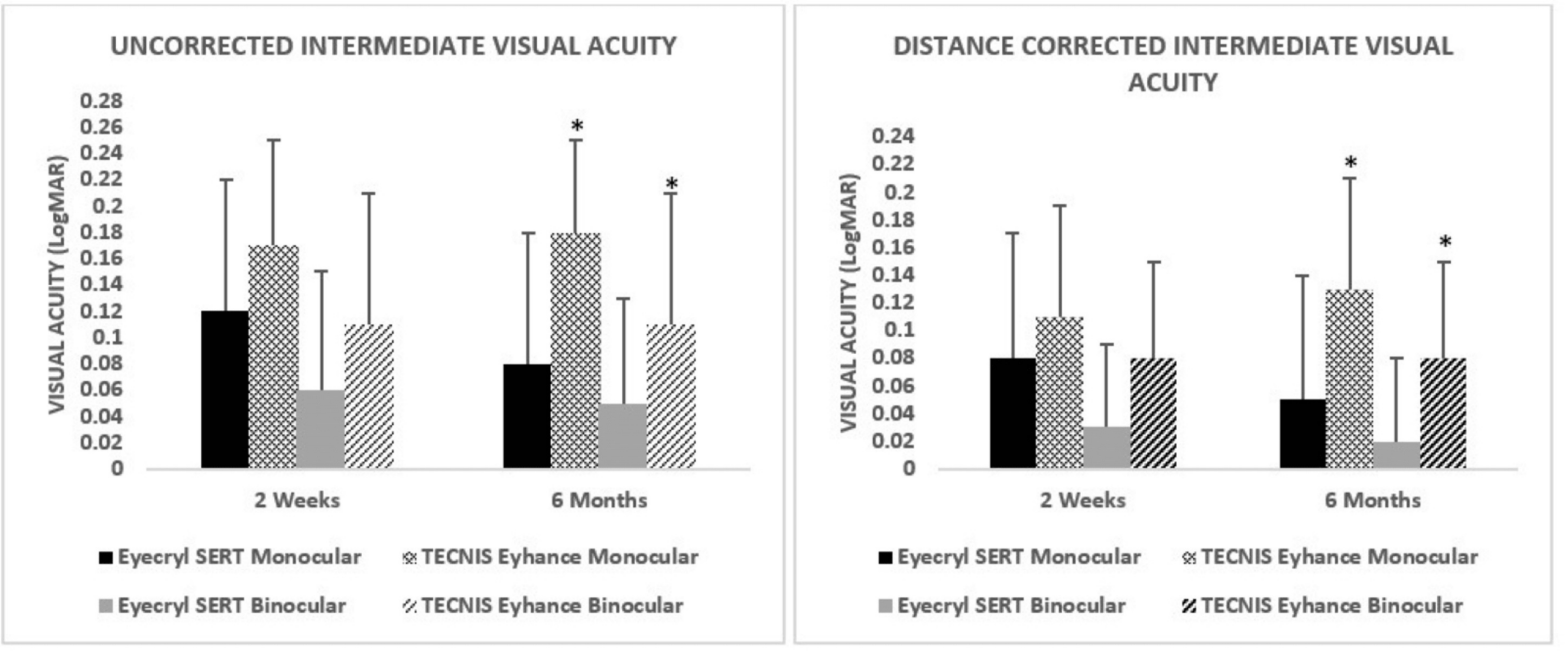
Comparison of UCIVA and DCIVA (logMAR) at two weeks and six months postoperatively in patients implanted with Eyecryl SERT and TECNIS Eyhance ^*^ A p-value ≤ 0.05 was considered statistically significant between groups. DCIVA, distance-corrected intermediate visual acuity; UCIVA, uncorrected intermediate visual acuity

For monocular DCIVA at the same distance, Eyecryl SERT improved from 0.08 ± 0.09 logMAR at two weeks to 0.05 ± 0.09 logMAR at six months (mean change: -0.03 ± 0.05 logMAR; p < 0.0001). TECNIS Eyhance showed a slight deterioration from 0.11 ± 0.08 to 0.13 ± 0.08 logMAR (mean change: +0.02 ± 0.05 logMAR; p = 0.017). Between-group analysis at six months again demonstrated a significant advantage with Eyecryl SERT (p < 0.0001) (Figure 1).

UCIVA at 66 cm

For monocular UCIVA at 66 cm, the Eyecryl SERT group demonstrated a statistically significant improvement, from 0.12 ± 0.10 logMAR at two weeks to 0.08 ± 0.10 logMAR at six months (mean change: -0.04 ± 0.05 logMAR; p < 0.0001). In contrast, the TECNIS Eyhance group showed a slight decline, with values increasing from 0.17 ± 0.08 logMAR at two weeks to 0.18 ± 0.07 logMAR at six months (mean change: +0.02 ± 0.04 logMAR; p = 0.0063). Between-group comparison at six months revealed a statistically significant advantage for Eyecryl SERT (p < 0.0001).

For binocular UCIVA at 66 cm, Eyecryl SERT showed a minor, nonsignificant improvement from 0.06 ± 0.09 logMAR at two weeks to 0.05 ± 0.08 logMAR at six months (mean change: -0.01 ± 0.03 logMAR; p = 0.125). TECNIS Eyhance remained unchanged at 0.11 ± 0.10 logMAR across both visits (p = 0.625). Nevertheless, inter-group comparison at six months demonstrated a statistically significant difference favoring Eyecryl SERT (p = 0.0009) (Figure 1).

Contrast sensitivity

**Figure 2:**
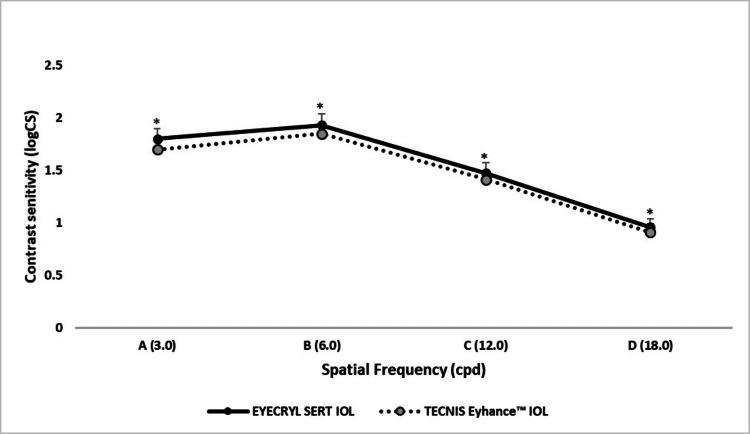
Comparative analysis of contrast sensitivity between Eyecryl SERT and TECNIS Eyhance groups ^*^ A p-value ≤ 0.05 was considered statistically significant between groups. IOL, intraocular lens

Across all tested spatial frequencies (3.0-18.0 cpd), Eyecryl SERT consistently demonstrated superior log contrast sensitivity compared to TECNIS Eyhance, with statistically significant differences at each frequency. Log contrast sensitivity values ranged from 0.96 to 1.93 for Eyecryl SERT and 0.91 to 1.85 for TECNIS Eyhance, favoring Eyecryl SERT across the spectrum. The advantage was particularly evident at mid-to-high spatial frequencies (6.0, 12.0, and 18.0 cpd), which are critical for tasks requiring fine visual discrimination, such as reading and facial recognition (Figure 2).

Defocus curve

**Figure 3:**
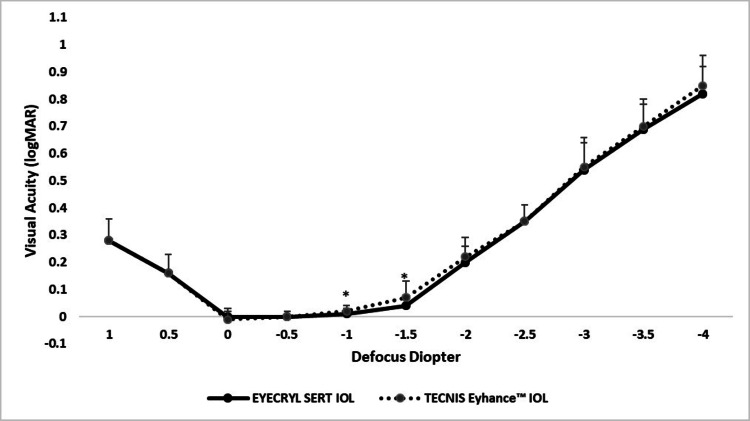
Binocular defocus curve comparing visual acuity (logMAR) across defocus levels for patients implanted with Eyecryl SERT and TECNIS Eyhance IOLs ^*^ A p-value ≤ 0.05 was considered statistically significant between groups. IOL, intraocular lens

The binocular defocus curve analysis demonstrated comparable visual acuity across most levels, with statistically significant differences observed at select intermediate defocus points. At +1.0 D and +0.5 D, both IOLs achieved equivalent visual performance (0.28 ± 0.08 and 0.16 ± 0.07 LogMAR, respectively; p > 0.05). At 0.0 D, Eyecryl SERT showed slightly better acuity (-0.02 ± 0.03 vs. -0.01 ± 0.02 LogMAR; p = 0.13), though the difference was not statistically significant. At -1.0 D and -1.5 D, Eyecryl SERT demonstrated superior performance (0.01 ± 0.02 vs. 0.02 ± 0.02 LogMAR; p = 0.05 and 0.04 ± 0.04 vs. 0.07 ± 0.06 LogMAR; p = 0.009). No statistically significant differences were observed at -0.5 D, -2.0 D, or beyond (p > 0.05), where the curves converged with minimal variation (Figure 3).

Reading speed

**Figure 4:**
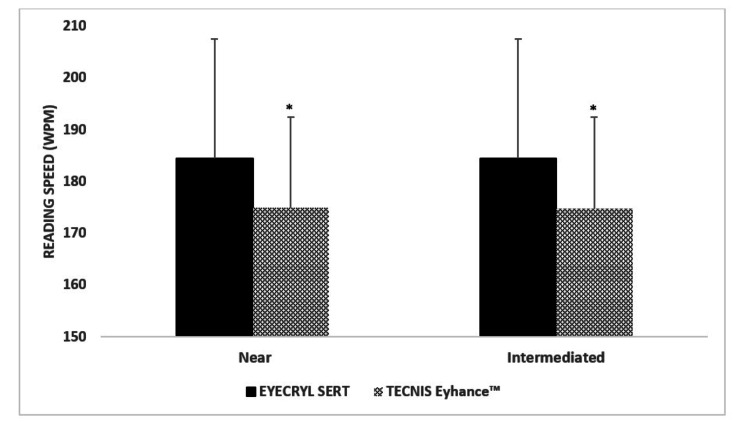
Comparison of reading speed (WPM) at near and intermediate distances in patients implanted with Eyecryl SERT and TECNIS Eyhance ^*^ A p-value ≤ 0.05 was considered statistically significant between groups. WPM, words per minute

Reading performance was evaluated at six months postoperatively using standardized SRD metrics. In the Eyecryl SERT group, the mean ± SD preferred reading distance was 48.3 ± 3.1 cm for near and 54.75 ± 3.05 cm for intermediate distances, with corresponding reading speeds of 184.4 ± 23.12 words per minute (WPM) for both distances. In comparison, the TECNIS Eyhance group demonstrated a preferred near reading distance of 50.1 ± 2.8 cm and an intermediate distance of 56.57 ± 2.57 cm, with reading speeds of 174.8 ± 17.53 WPM for both distances. Differences between groups were statistically significant for both near and intermediate reading speeds (p = 0.0074), favoring Eyecryl SERT (Figure 4).

Subjective questionnaire

Postoperative evaluation using the Vision-Related Quality of Life questionnaire demonstrated significant improvement in both groups, with high overall satisfaction scores at six months (Eyecryl SERT: 89.73 ± 0.55; TECNIS Eyhance: 89.53 ± 1.41; p = 0.7992), indicating comparable patient-perceived visual quality.

Nondirected optical/visual symptoms

At both postoperative Day 1 and Month 6, none of the subjects in the Eyecryl SERT group reported any spontaneously experienced ocular or visual symptoms in response to nondirected questioning. In the TECNIS Eyhance group, three patients reported mild glare and halos on Day 1, which were transient and resolved without intervention. These symptoms were self-reported, subjective in nature, and did not affect visual acuity or safety outcomes, suggesting a high level of visual comfort and absence of significant photic phenomena following implantation.

PCO assessment

No cases of PCO were reported in either group during the study period.

Adverse event

No adverse events were observed in either group during the study period.

## Discussion

The present study is part of a large prospective, interventional, comparative investigation that demonstrated better or comparable visual performance of Eyecryl SERT relative to TECNIS Eyhance across multiple parameters over a six-month postoperative period. Both cohorts were demographically similar, with balanced gender distribution and mean ages of 67.3 ± 8.0 years (Eyecryl SERT) and 65.9 ± 7.4 years (TECNIS Eyhance) (p > 0.05).

Regarding DCIVA at 66 cm, Eyecryl SERT consistently showed improvement in both monocular and binocular assessments, with statistically significant advantages at six months. Specifically, monocular DCIVA at 66 cm improved by -0.03 ± 0.05 LogMAR in the Eyecryl SERT group, compared to a slight worsening (+0.02 ± 0.05 LogMAR) in the TECNIS Eyhance group (p < 0.0001). These results are consistent with published data reporting mean monocular DCIVA at six months of 0.26 ± 0.08 LogMAR for Tecnis Symfony OptiBlue (DXR00V) and 0.23 ± 0.06 LogMAR for Tecnis Synergy (DFR00V) IOLs, values that align well with the 0.05 ± 0.09 LogMAR (Eyecryl SERT) and 0.02 ± 0.05 LogMAR (Eyhance) reported in this study [11]. Similarly, Bernabeu-Arias et al. (2023) showed that the ISOPURE 123 IOL yielded binocular DCIVA of 20/25 or better (≤0.1 LogMAR) in 80.65% of patients at 66 cm, further supporting the efficacy of IOLs optimized for intermediate vision [12].

For UCIVA Eyecryl SERT also demonstrated better binocular outcomes at 66 cm at six months, with mean changes of -0.01 ± 0.03 LogMAR and 0.00 ± 0.03 LogMAR, respectively, compared to +0.01 ± 0.04 and -0.01 ± 0.03 LogMAR in the TECNIS Eyhance group (p = 0.0009 and p = 0.0005, respectively). Although monocular improvements were less pronounced, they followed a similar trend. Dell et al. (2024) reported a monocular UIVA of 0.23 ± 0.18 LogMAR at 66 cm one month after TECNIS Eyhance implantation, comparable to UCIVA values of 0.12 ± 0.10 LogMAR (SERT) and 0.17 ± 0.08 LogMAR (Eyhance) in the current study at two weeks [13]. Likewise, Auffarth et al. (2021) observed six-month monocular UCIVA and DCIVA values of 0.16 ± 0.02 and 0.19 ± 0.02 LogMAR, respectively, and binocular values of 0.07 ± 0.12 and 0.09 ± 0.11 LogMAR, respectively, following TECNIS Eyhance implantation [14].

Assessment of reading speed (SRD) at six months also favored Eyecryl SERT. At the preferred near distance, Eyecryl SERT achieved 48.3 ± 3.1 cm compared to 50.1 ± 2.8 cm for TECNIS Eyhance (p = 0.0010), with corresponding reading speeds of 184.38 ± 23.12 WPM and 174.83 ± 17.53 WPM, respectively (p = 0.0074). At intermediate distances, the preferred working distance was 54.75 ± 3.05 cm (Eyecryl SERT) and 56.57 ± 2.57 cm (TECNIS Eyhance; p = 0.0017), with respective reading speeds of 184.4 ± 23.12 WPM and 174.8 ± 17.53 WPM (p = 0.0074). These results are slightly better than those reported for the EDOF IOL (Evolve, Soleko, Rome, Italy), which demonstrated a mean reading speed of 152.1 ± 48.2 WPM (range: 92-243 WPM) at six months follow-up [15]. Collectively, these findings reinforce the advantage of Eyecryl SERT in delivering enhanced intermediate visual function, reading performance, and contrast sensitivity, without compromising distance acuity or safety.

The subjective questionnaire demonstrated significant postoperative improvement in both groups, with high overall satisfaction scores at six months (Eyecryl SERT: 89.73 ± 0.55; TECNIS Eyhance: 89.53 ± 1.41; p = 0.7992), indicating similar levels of patient-perceived visual quality. Contrast sensitivity testing under photopic conditions consistently favored Eyecryl SERT across all spatial frequencies. At 3.0 cycles per degree (cpd), Eyecryl SERT achieved a mean sensitivity of 1.80 ± 0.10 compared to 1.70 ± 0.10 for TECNIS Eyhance (p < 0.0001). Similarly, at 6.0 cpd, values were 1.93 ± 0.10 for Eyecryl SERT versus 1.85 ± 0.11 for TECNIS Eyhance (p = 0.0004); at 12.0 cpd, 1.475 ± 0.085 vs. 1.415 ± 0.100 (p = 0.0021); and at 18.0 cpd, 0.96 ± 0.08 vs. 0.91 ± 0.08 (p = 0.0004). These findings underscore the enhanced performance of Eyecryl SERT in low-contrast environments and are consistent with outcomes reported for the TECNIS Symfony IOL in prior studies [16,17].

Defocus curve analysis revealed comparable visual acuity profiles between both IOLs across the full range of vergences, with statistically significant advantages for Eyecryl SERT at specific focal points. At 0.0 D (distance vision), Eyecryl SERT showed slightly better acuity (-0.02 ± 0.03) compared to TECNIS Eyhance (-0.01 ± 0.02; p = 0.0108), and at -1.0 D (intermediate range), Eyecryl SERT again outperformed TECNIS Eyhance (0.01 ± 0.02 vs. 0.02 ± 0.02; p = 0.0091), reflecting enhanced performance in daily visual tasks. These trends align with previous findings for TECNIS Symfony IOLs [16].

A literature survey reported a PCO rate of 10% following Tecnis Symfony implantation at six months, compared to 0% in the current study during the same follow-up period [18]. Additionally, no adverse events or vision-related symptoms were reported, confirming the safety and optical stability of both lenses. Eyecryl SERT consistently demonstrated a modest but meaningful advantage in visual function, particularly in intermediate vision and contrast sensitivity, over TECNIS Eyhance.

Limitations

This study has several limitations. Being a single-center study, the results may not be broadly applicable across diverse populations or surgical practices. The six-month follow-up period limits the evaluation of long-term outcomes, including refractive stability, PCO development, and durability of visual improvements. The sample size was small, and while the observed outcomes are encouraging, confirmation through larger, multicenter studies with extended follow-up would further strengthen the evidence base and support wider generalizability.

## Conclusions

This study demonstrates that Eyecryl SERT provides better or comparable visual performance relative to the TECNIS Eyhance IOL across measures of intermediate visual acuity, contrast sensitivity, reading speed, and defocus range over a six-month postoperative period. Both IOLs exhibited excellent safety profiles, stable refractive outcomes, and high patient satisfaction. These findings support the clinical utility of Eyecryl SERT as an effective option for enhancing intermediate vision while maintaining high-quality distance vision and contrast sensitivity in patients undergoing cataract surgery. Further long-term and larger-scale studies are warranted to confirm these results and establish broader clinical relevance.
